# Effectiveness and safety assessment of drospirenone/ethinyl estradiol tablet in treatment of PCOS patients: a single center, prospective, observational study

**DOI:** 10.1186/s12905-020-00905-x

**Published:** 2020-02-27

**Authors:** Li Li, Ruiqin Zhang, Jing Zeng, Hu Ke, Xiuhong Peng, Liying Huang, Hongmei Zhang, Zhijing Chen, Tian Tian Li, Qiuxiao Tan, Ying Yang, Xiaofang Li, Xin Li

**Affiliations:** 1 Guangdong Women and Children Hospital, Guangzhou, 510010 China; 2 Guangzhou Medical University, Guangzhou, 510182 China; 3 Obstetrics and Gynecology Hospital of Fudan University, Shanghai, 200011 China

**Keywords:** Drospirenone, Ethinyl estradiol, PCOS, COC

## Abstract

**Background:**

To investigate the effectiveness and safety of 3 mg drospirenone and 20 μg ethinyl estradiol tablet (3 mg DRSP/20 μg EE) in the treatment of polycystic ovary syndrome (PCOS).

**Methods:**

This single center, prospective observational study was conducted in 140 patients with PCOS. They were prescribed 3 mg DRSP/20 μg EE in a 24/4/ regimen for 3 months. Patients were instructed to take oral DRSP/EE tablets (once daily) on the 2nd day of menstruation, for 28 consecutive days for 1 cycle. After 3 months of treatment, anthropometric assessments along with variations in sex hormones related index, glucolipid metabolic index, changes in bilateral ovarian volume, as well as adverse effect of the combination were evaluated.

**Results:**

When compared to baseline, body mass index (BMI, 22.07 ± 4.09 vs. 21.35 ± 3.22, *p* < 0.001) and waist hip ratio (WHR, 0.86 ± 0.07 vs. 0.854 ± 0.06, *p* = 0.026) decreased significantly after treatment. Sex-hormones such as luteinizing hormone (LH) (10.88 vs. 5.81 U/L), testosterone (T) (1.85 vs. 1.51 nmol/L) and free androgen index (FAI) (5.37 vs. 1.50) decreased significantly after treatment (*p* < 0.001). Follicular stimulating hormone (FSH) increased significantly at 3 months as compared to before treatment (5.13 vs. 5.42 U/L, *p* = 0.009). Plasma insulin (11.03 vs. 11.10 pmol/L), fasting (4.97 vs. 4.93 mmol/L) and 2 h-blood glucose levels (7.18 vs. 7.04 mmol/L) did not change when compared to baseline. Plasma triglycerides (TG, 1.32 vs. 1.65 mmol/L) significantly increased 3 months after treatment when compared to before treatment (*p* < 0.001). However, high density lipoprotein-cholesterol (HDL-C) levels increased significantly after treatment (1.41 vs. 1.57 mmol/L, *p* < 0.001). It was seen that, when compared to baseline, bilateral ovarian volume (left and right) was significantly lower after treatment (*p* < 0.05). It was seen that 81 patients reported no adverse reactions. Of the common discomforts reported, breast swelling and pain, gastrointestinal disorder and dizziness and headache were most frequent.

**Conclusions:**

Treatment of PCOS patients with3 mg DRSP/20 μg EE has shown beneficial hormonal and lipid profile along with considerable safety profile.

**Trial registration:**

Chinese Clinical Trial Registry ChiCTR1900022001, March 2019, retrospectively registered.

## Background

Polycystic ovarian syndrome (PCOS) is a well-known endocrine disorder in women of reproductive age [1]. PCOS is a syndrome of ovarian dysfunction, with its hallmark features being hyperandrogenism and polycystic ovarian morphology [2], greatly impacting a woman’s reproductive life [3]. Nevertheless, the clinical manifestations may also include menstrual irregularities, metabolic dysfunction and obesity [4].

It has also been observed that approximately 60–80% of PCOS patients have insulin resistance, and 95% of obese patients are with increased risk for cardiovascular disease and type 2 diabetes [5]. According to the Rotterdam PCOS criteria, the prevalence of PCOS in the Chinese Han population is 5.6% [6].

Owing to the complex pathophysiology of PCOS, laying specific guidelines for its management has posed immense challenge to policy makers and the approach varies widely between the endocrinologists, gynecologists, and dermatologists [7]. Previous reports have given conflicting results about the clinical, hormonal, and reproductive outcomes [8, 9]. The observed variation in the results is a source of confusion that prompted the researchers to look at alternate therapeutic options.

At present, combined oral contraceptive (COC) is an effective method for the treatment of PCOS. Use of fourth-generation COC containing ethinylestradiol (EE) together with a novel progestin, drospirenone (DRSP), have claimed to have properties closer to those of natural progesterone, including anti-mineralocorticoid and antiandrogenic activities [10]. The progesterone component of DRSP/EE tablets is a steroid 17a-spironolactone derivative, with strong progesterone-like, salt-corticosteroid resistant and anti-androgen pharmacological activity. The introduction of this novel COC into clinical practice has been unprecedentedly swift, but its effects in adolescents and young women with PCOS are unknown. The goal of this approach was to obtain a regular menstrual cycle and to improve the clinical signs of hyperandrogenism [10].

However, available literature clearly points at the increased risk of venous and arterial thrombosis associated with long-term use of oral contraceptives [11]. It has also been documented that the risk of venous thrombosis increases with higher EE dose [12, 13]. In this regard, the international evidence-based guideline for the assessment and management of PCOS 2018 recommend that low estrogen dose COC (20–30 μg of EE) is a better first-line treatment program [14] which is offered in 3 mg DRSP/20 μg EE combination.

The purpose of this study was therefore to investigate the effectiveness of 3 mg DRSP/20 μg EE in treating PCOS patients in terms of cardiometabolic risk factors, sex hormone related variation and lipid parameters including fasting blood glucose and insulin levels. Additionally, effects on ovarian volume and the adverse effects were also assessed.

## Methods

### Study population

This single center, prospective observational study was conducted from August 2017 to December 2018. Among the 173 patients with PCOS who were enrolled from the Department of Gynecology, Guangdong Maternal and Child Health Hospital, only 140 patients completed the study treatment protocol. The study was approved by Institutional ethical committee of Guangdong Maternal and Child Health Hospital. The study was conducted in accordance with the Good Clinical Practice guidelines and Declaration of Helsinki.

Considering that the prevalence of PCOS is 6–10%, a minimum sample size of 122 was required at a 1% level of significance with a power of 90%. However in order to account for any attrition, a total of 140 patients were enrolled.

Patients who qualified for PCOS as per the European Society of Human Reproduction and Embryology and the American Society of Reproductive Medicine at the Rotterdam Conference in 2003 [4] were included. Additionally, patients with no contraindication to oral contraceptives, who were confident of being compliant to the drug and provided informed consent were also included. On the other hand, patients with any contraindications to oral contraceptives; desirous of conception within 6 months of inclusion in the study; diagnosed with concomitant hypothyroidism, hyperprolactinemia, diabetes mellitus, renal, or adrenal insufficiency; history of drug use for PCOS before inclusion in the study, and history of smoking and drug abuse were excluded from the study.

### Drugs and treatment

The patients at clinician’s discretion received DRSP/EE tablets (Yousiyue, Bayer Medical and Health Company, Import Drug Registration No. H20140972). Each strip contained 28 tablets; 24 active tablets and 4 placebo tablets. Every active tablet contained 20 μg of EE and 3 mg of DRSP. Patients were instructed to take oral DRSP/EE tablets (once daily before bedtime) on the 2nd day of menstruation or on the second day of withdrawal bleeding, for 28 consecutive days for 1 cycle. There were 3 consecutive treatment cycles and patients were followed up for 3 months of treatment.

Although, till date no established evidence exists whether a short- or a long-term usage of COC is beneficial in terms of efficacy and safety benefits, it is considered best to review the effects after 3 months. Most studies show that unscheduled bleeding is more likely in the first cycle, and some show an improvement over the first 3 months, with the incidence of unscheduled bleeding remaining constant from 4 to 12 months for OC in Chinese women [15]. Thus, it is recommended that women experiencing unscheduled bleeding continue their method for at least 3 months before seeking advice. With this background, we set up a 3 month trial period to assess the effect of 3 mg DRSP/20 μg EE in PCOS patients for 3 months.

### Study measures

Baseline data were collected from the patients including demographic details such as age and presenting complaints (menstrual history, drug history, etc.). Patient’s weight was recorded using a digital scale, height using a stadiometer (Guangzhou Quality and Technical Supervision Bureau Guangzhou Institute of Metrology and Measurement Technology Measuring Instruments No. 794067), waist-hip ratio (WHR) was measured using a standard measuring tape and body mass index (BMI) was calculated. Additionally, hormonal profile [leutinizing hormone (LH), follicular stimulating hormone (FSH), testosterone (T), free androgen index (FAI), androgen] and biochemical parameters[fasting plasma glucose (FPG), 2-hourblood glucose (oral glucose tolerance test using 83 g glucose in 250–300 ml water), fasting insulin (FINS), total cholesterol (TC), triglycerides (TG), high density lipoprotein-cholesterol (HDL-C), low density lipoprotein-cholesterol (LDL-C)] were measured using fasting blood samples followed by an overnight fast. Variation in Homeostasis model assessment of insulin resistance (HOMA-IR) was also assessed at end of treatment regimen [16].Ovarian volume was measured by gynecological ultrasonography (Samsung, specification model: H60, host serial number: S10LM3HHB00009A). After 3 months of medication, the above indicators were reviewed and compared. Similarly, bleeding pattern during medication and presence of adverse reactions (ADRs) were also recorded.

Menstrual bleeding volume was categorized into drip, small, normal and large amount of bleeding. Drip bleeding was defined when the need of sanitary napkin was not required. When the volume was lesser than the normal menstrual bleeding, it was considered as small amount of bleeding and normal was when the bleeding volume was similar to normal menstrual cycle. Large amount of bleeding was defined as volume more than the regular cycle. As recommended by WHO, the 90 days was considered as a reference period to calculate the amount / number of days and times of drip bleeding.

### Statistical analysis

All statistical analyses were performed using SPSS16.0 software. Missing data were excluded from the analysis. Continuous data were expressed as mean ± standard deviation and categorical data were expressed as percentages (%).25th, 50th, and 75th percentiles were also calculated. If the normal distribution was not met for Student’s t-test, the signed rank sum test of the paired design data was used and the difference was considered statistically significant at *p* < 0.05.

## Results

### Baseline characteristics

At baseline, it was seen that all patients (mean age: 24.64 ± 4.31 years) had normal BMI (22.07 ± 4.09 kg/m^2^) and WHR (0.86 ± 0.07, Table 1). The lipid profile was in normal range for all parameters and FPG was 4.97 ± 0.43 mmol/L. At baseline, FINS levels were 11.06 ± 6.23 pmol/L and HOMA-IR was 2.50 ± 1.64.

**Table 1:** Baseline characteristics of study patients

Variables	n	Endpoint	Mean ± SD	25th	50th	75th	p
Age (years)	140	–	24.65 ± 4.33	22.00	25.00	27.75	–
BMI (kg/m^2^)	140	Before	22.07 ± 4.09	19.00	21.30	24.75	< 0.001
		After	21.35 ± 3.22	18.92	21.18	23.04	
WHR	138	Before	0.86 ± 0.07	0.82	0.86	0.90	0.026
		After	0.85 ± 0.06	0.81	0.85	0.89	
LOV (cm^3^)	49	Before	11.80 ± 10.66	6.73	9.45	12.78	< 0.001
		After	7.46 ± 3.21	5.13	6.98	9.33	
ROV (cm^3^)	48	Before	12.31 ± 10.49	7.71	9.33	12.02	0.001
		After	9.02 ± 6.24	5.30	7.72	10.62	

*BMI* body mass index; *WHR* waist-hip ratio; *LOV* left ovarian volume; *ROV* right ovarian volume

### Metabolic and hormonal profile

When compared to baseline, anthropometric parameters such BMI and WHR decreased significantly with treatment at 3 months follow up (Table 1).

**Fig. 1:**
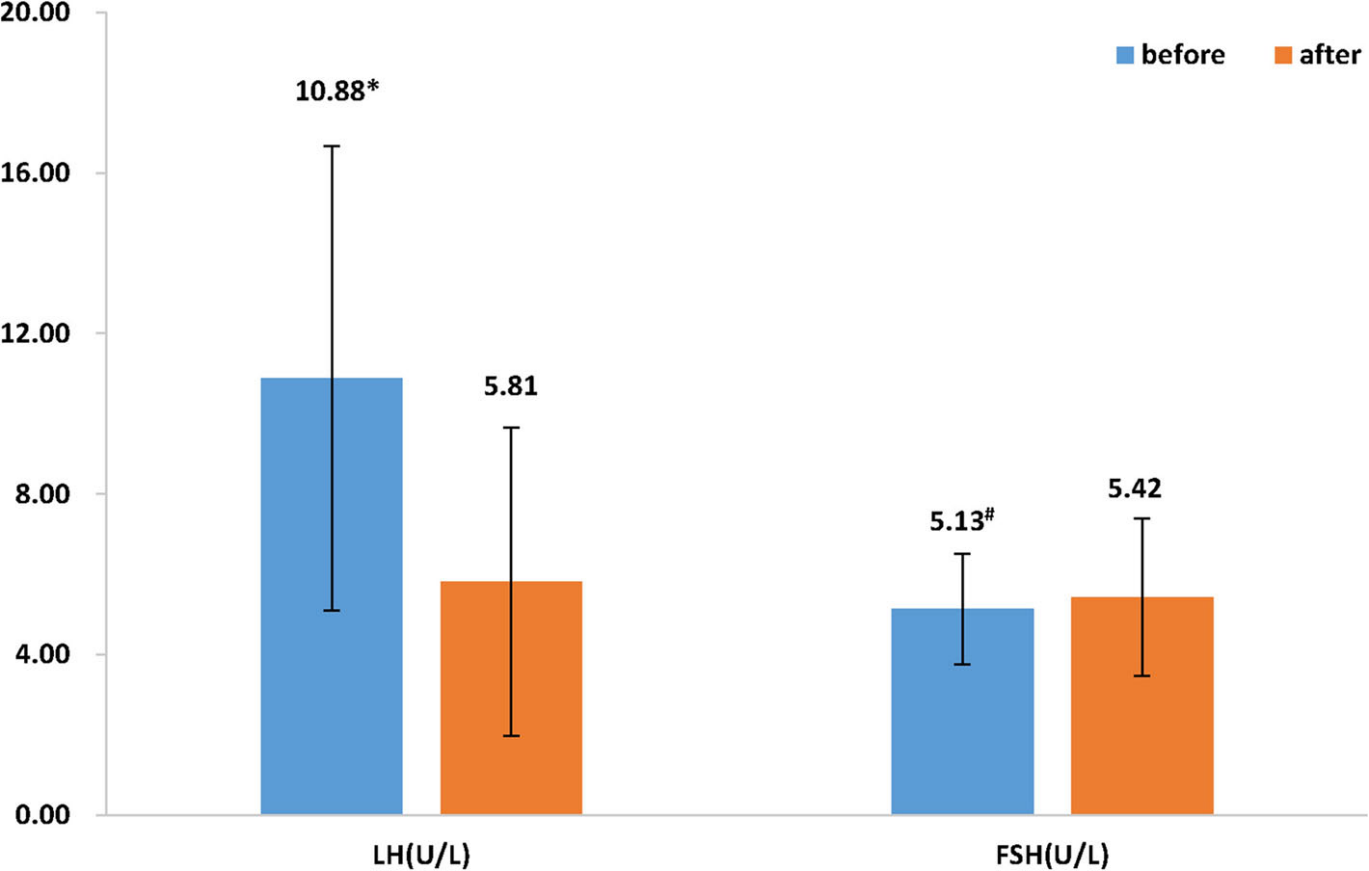
Comparison of LH and FSH before and after treatment

**Fig. 2:**
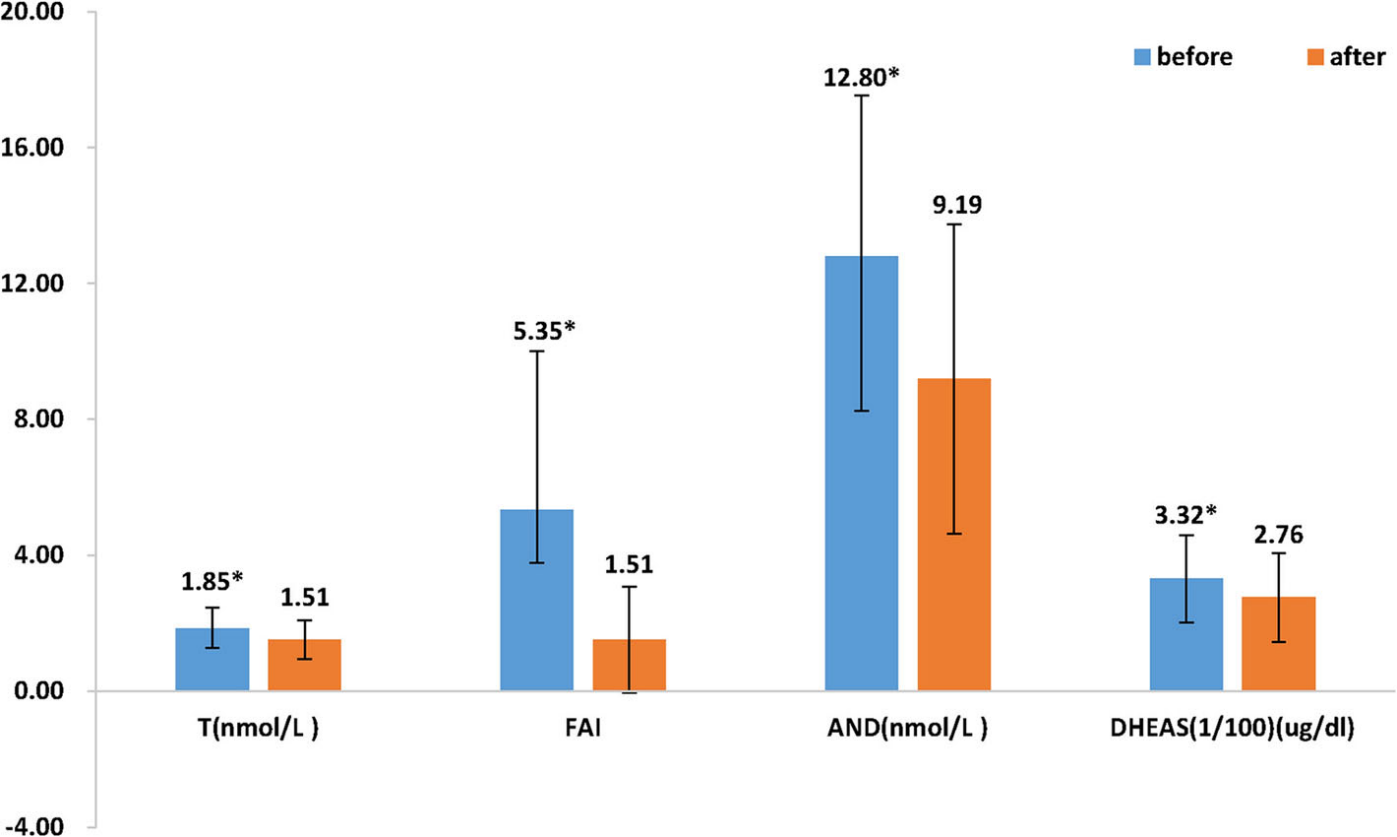
Comparison hormonal profile before and after treatment

The levels of LH (10.88 vs. 5.81 U/L), T (1.85 vs. 1.51 nmol/L), FAI (5.35 vs. 1.51) and androgen (12.80 vs. 9.19 nmol/L) in PCOS patients decreased significantly after treatment when compared to before treatment (*p* < 0.001) (Figs. 1 and 2). FSH increased significantly at follow up as compared to before treatment (5.13 vs. 5.42 U/L, *p* = 0.009, Fig. 1).

**Fig. 3:**
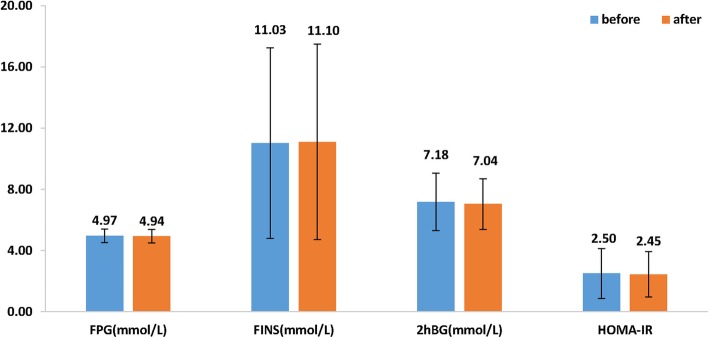
Comparison of glucose assessment parameters before and after treatment

**Fig. 4:**
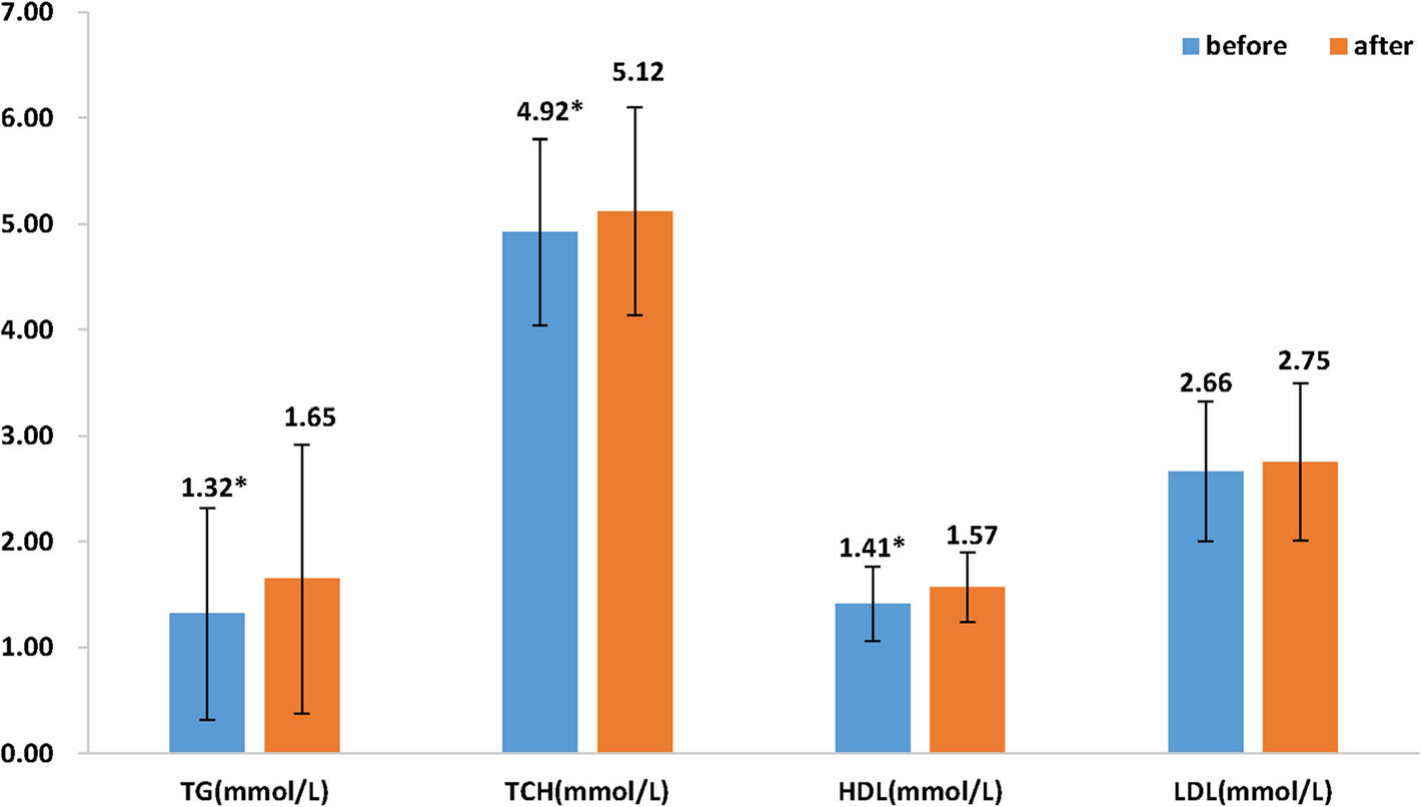
Comparison of lipid parameters before and after treatment

It was seen that plasma insulin (11.03 vs. 11.10 pmol/L), fasting (4.97 vs. 4.93 mmol/L) and 2 h-blood glucose levels (7.18 vs. 7.04 mmol/L) did not change after 3 months of treatment as compared to baseline. HOMA-IR also remained unchanged after treatment (Fig. 3). In terms of lipid profile, plasma TG (1.32 vs. 1.65 mmol/L; *p* < 0.001) significantly increased 3 months after treatment whereas TC (4.92 vs. 5.12 mmol/L) increased when compared to before treatment. However, HDL-C levels significantly increased after treatment (1.41 vs. 1.57 mmol/L, *p* < 0.001), Fig. 4.

When compared to baseline, bilateral ovarian volume (left and right) was significantly lower after treatment (*p* < 0.05, Table 1).

### Safety events

It was seen that 138 patients (98.57%) had withdrawal bleeding. Among them, 3 (2.14%) had spotting, 23 (16.43%) had less menstrual bleeding, 113 (80.71%) had normal menstruation and 1 (0.71%) had excessive menstrual bleeding, Table 2.

**Table 2:** Bleeding patterns and ADRs in PCOS patients treated with DRSP/EE

	n (%)
***Bleeding pattern***	
Withdrawal bleeding	138 (98.57)
Spotting	3 (2.14)
Less menstruation	23 (16.43)
Normal menstruation	113 (80.71)
***ADRs***	
No ADRs	81 (57.86)
Nausea	4 (2.16)
Headache and dizziness	4 (2.86)
Breast pain	20 (14.29)
Irregular bleeding	50 (35.71)
Others	1 (0.71)

*ADRs* adverse reactions

Among the 140 PCOS patients who received 3 mg DRSP/20 μg EE tablet, it was seen that 81 (57.86) reported no adverse reactions. However, 58 (42.14%) patients had discomfort, including 1 (0.72%) case of gastrointestinal disorder, 4 (2.86%) dizziness and headache, 20 (14.29%) breast swelling and pain cases. It was seen that 50 (35.71) patients had irregular bleeding, of which 3 cases were caused due to non-compliance, Table 2.

## Discussion

Management of PCOS is generally aimed at regularizing menstrual cycles, suppression of ovarian T production and regulate impaired metabolic risk factors. Conventionally, this is achieved using COC. Nevertheless, severe side effects associated with long-term use of COC include increased risk of venous thrombosis and weight gain, making the use of COC inappropriate in PCOS therapy [17]. Thus, this study assessed the effectiveness of oral contraceptive containing 3 mg DRSP/20 μg EE in Chinese patients with PCOS.

The present study results show that 3 mg DRSP/20 μg EE combination is safe in non-obese PCOS patients along with overall beneficial effects on the metabolic and hormonal profile. Variations in lipid profile during COC intake usually depends on the dose and androgenic activity of EE and progestogens with androgenic activity is known to shift lipid and lipoprotein metabolism in a potentially unfavorable way [18-21].Similarly, a significant increase in total TG was seen in the current study that is similar to that reported elsewhere [16, 17]. It has been demonstrated that COC caused increase in TGs, a potential risk factor for cardiovascular disease is due to increased lipoprotein synthesis and not due to impaired lipolytic catabolism [22], an underlying risk factor for atherogenic process [23]. Additionally, it should be noted that the COC-induced elevation of TG levels is mainly due to an increased production of VLDL which is eliminated via receptor-mediated process in hepatocytes as stimulated by EE and are not converted into small VLDL or LDL [22]. Furthermore, the measured TG values in the current study were still well within the accepted ranges for TG levels. Clinically relevant values for increased risk for coronary heart disease are > 150 mg/dL for TG and > 200 mg/dL for TC [24].

In this study, higher levels of HDL-C were found in patients using 3 mg DRSP/20 μg EE (Table 1). This finding is in line with the literature [25] and shows that despite of the presence of the progestogen, the levels of HDL-C were higher. On the contrary, a study developed with 48 women treated with contraceptive containing androgenic progestogen (levonorgestrel/20EE) observed decrease in HDL-C levels in comparison to basal analyses [26].

DRSP, the only progestin with aldosterone antagonist activity, is known to mitigate the aldosterone induced insulin resistance by inhibiting biosynthesis and affinity of insulin receptors [27]. Thus, it could be hypothesized that the role of maintaining insulin homeostasis and glucose levels could be ascribed at least in part to the DRSP–aldosterone antagonist activity per se. Our study also found that DRSP/EE combination in PCOS patients did not alter fasting glucose, 2 h-blood glucose or insulin level even after 3 months of treatment (Table 1). In other words, insulin sensitivity was well maintained as seen in other studies [28] and that DRSP/EE combination might be considered neutral in its effect on insulin resistance, a major player of PCOS [14, 16]. Although studies in Europe suggest the combination of EE (35 μg EE) with 2 mg cyproterone acetate is widely used COC for PCOS [29], the present study shows that the EE/DRSP can be a reasonable alternative for Chinese PCOS patients.

Hyperandrogenism and polycystic ovaries leading to ovarian dysfunction are the hallmark features of PCOS [4]. The combination of DRSP/EE has been demonstrated to directly reduce the synthesis of T and its precursors in the ovary [10]. In agreement, we also observed a significant reduction in plasma T and androgen levels post therapy with 3 mg DRSP/20 μg EE (Table 1). By inhibiting FSH and LH, estrogen blocks the formation and maturation of ovarian follicles thereby suppressing ovulation [30]. Moreover, COCs causes increase in concentration of sex-hormone binding globulin (SHBG), thus reducing free T levels and thereby causing androgen deprivation [31]. A higher FAI and T levels were seen at baseline which reduced significantly after treatment in our PCOS patients. We also found a less pronounced, yet significant decrement in BMI and WHR (Table 1).

In PCOS patients with no reproductive requirements, the long-term use of COCs have shown to increase the risk of venous thrombosis. However, none of the study patients reported venous thromboembolism (VTE) but milder ADRs that included breast tenderness/swelling along with headache and dizziness as reported elsewhere [32]. The degree of cycle control was also effective with majority of women having normal menstruation at follow up (Table 2).

Although benefits of 3 mg DRSP/20 μg EE in PCOS patients is established in the current study, this was a retrospective, observational design with a small sample size being considered. As a future prospect, a prospective randomized controlled trial has been further planned with larger sample size to demonstrate the efficacy and safety of 3 mg DRSP/20 μg EEalong with its clinical application in patients with PCOS. Finally, since the study was retrospectively registered, it could have induced a bias to the reported findings.

On the other hand, an important strength of this study is its crossover design, in which the intervention’s effect was evaluated within the same patients, eliminating between-subject variability.

## Conclusion

Results of the present study do not support any harmful effect of all 3 mg DRSP/20 μg EE in women with PCOS. In particular the present data show that in non-obese women with PCOS, 3 mg DRSP/20 μg EE improves the metabolic and hormonal profile of these women. Further research is needed to evaluate if these results may be extended to obese women with PCOS with severe metabolic derangement.

## Data Availability

The dataset analyzed during this study is available from the corresponding author on reasonable request.

## Abbreviations

ADRs: Adverse Reactions
BMI: Body Mass Index
COC: Combined Oral Contraceptive
DRSP: Drospirenone
EE: Ethinyl Estradiol
FAI: Free Androgen Index
FINS: Fasting Insulin
FPG: Fasting Plasma Glucose
FSH: Follicular Stimulating Hormone
HDL-C: High Density Lipoprotein-Cholesterol
HOMA-IR: Homeostasis Model Assessment of Insulin Resistance
LDL-C: Low Density Lipoprotein-Cholesterol
LH: Leutinizing Hormone
PCOS: Polycystic Ovarian Syndrome
T: estosterone
TC: Total Cholesterol
TG: Triglycerides
VTE: venous thromboembolism
WHR: Waist-Hip Ratio
